# Feasibility, acceptability and efficacy of a text message-enhanced clinical exercise rehabilitation intervention for increasing ‘whole-of-day’ activity in people living with and beyond cancer

**DOI:** 10.1186/s12889-019-6767-4

**Published:** 2019-06-03

**Authors:** Sjaan R. Gomersall, Tina L. Skinner, Elisabeth Winkler, Genevieve N. Healy, Elizabeth Eakin, Brianna Fjeldsoe

**Affiliations:** 1 The University of Queensland, School of Health and Rehabilitation Sciences, Brisbane, QLD 4072 Australia; 2 The University of Queensland, School of Human Movement and Nutrition Sciences, Brisbane, Australia; 3 The University of Queensland, School of Public Health, Brisbane, Australia; 4 Baker Heart and Diabetes Institute, Melbourne, Australia; 5 Curtin University, School of Physiotherapy and Exercise Science, Perth, Australia

**Keywords:** Physical activity, Sedentary behavior, Use of time, Text messaging, Cancer survivors, Exercise oncology, Randomised controlled trial

## Abstract

**Background:**

Exercise interventions are typically delivered to people with cancer and survivors via supervised clinical rehabilitation. However, motivating and maintaining activity changes outside of the clinic setting remains challenging. This study investigated the feasibility, acceptability and efficacy of an individually-tailored, text message-enhanced intervention that focused on increasing whole-of-day activity both during and beyond a 4-week, supervised clinical exercise rehabilitation program for people with cancer and survivors.

**Methods:**

Participants (*n* = 36; mean ± SD age 64.8 ± 9.6 years; 44.1 ± 30.8 months since treatment) were randomized 1:1 to receive the text message-enhanced clinical exercise rehabilitation program, or the standard clinical exercise rehabilitation program alone. Activity was assessed at baseline, 4-weeks (end of the standard program) and 12-weeks (end of enhanced program) using both device (activPAL accelerometer; sitting, standing, light-stepping, moderate-stepping) and self-report [Multimedia Activity Recall for Children and Adults (MARCA); sedentary, light, moderate-to-vigorous physical activity (MVPA)] methods. The MARCA also assessed time use domains to provide context to activity changes. Changes and intervention effects were evaluated using linear mixed models, adjusting for baseline values and potential confounders.

**Results:**

The study had high retention (86%) and participants reported high levels of satisfaction [4.3/5 (±0.8)] with the intervention. Over the first 4 weeks, MARCA-assessed MVPA increased [+ 53.2 (95%CI: 2.9, 103.5) min/d] between groups, favoring the text message-enhanced program, but there were no significant intervention effects on sedentary behavior. By 12 weeks, relative to the standard group, participants in the text message-enhanced group sat less [activPAL overall sitting: − 48.2 (− 89.9, − 5.6) min/16 h awake; MARCA: -80.1 (− 156.5, − 3.8) min/d] and were participating in more physical activity [activPAL light stepping: + 7.0 (0.4, 13.6: min/16 h awake; MARCA MVPA: + 67.3 (24.0, 110.6) min/d]. The time-use domains of Quiet Time [− 63.3 (− 110.5, − 16.0) min/d] and Screen Time [− 62.0 (− 109.7, − 14.2) min/d] differed significantly between groups.

**Conclusions:**

Results demonstrate feasibility, acceptability and efficacy of a novel, text message-enhanced clinical exercise rehabilitation program to support changes in whole-of-day activity, including both physical activity and sedentary behavior. Changes were largely seen at 12-week follow-up, indicating potential for the intervention to result in continued improvement and maintenance of behavior change following a supervised exercise intervention.

**Trial registration:**

This trial is registered with the Australian New Zealand Clinical Trials Registry (ACTRN12616000641493; date registered 17/5/16).

**Electronic supplementary material:**

The online version of this article (10.1186/s12889-019-6767-4) contains supplementary material, which is available to authorized users.

## Background

Over one million Australians are now living beyond a diagnosis of cancer and ensuing treatment, with an estimated cost to the health care system of AU$1 million per incident case [1]. In this growing population, physical activity has been shown to improve disease- and treatment-related side effects, reduce the risk of cancer recurrence and common co-morbidities (cardio-metabolic diseases), and improve quality of life [2–5]. Face-to-face clinical exercise rehabilitation is a common mode of delivery for physical activity intervention among people with cancer and survivors [6]. However, despite short-term (i.e. < 12 weeks) increases in muscle strength, exercise capacity, physical function, flexibility and wellbeing, physical activity outside of the supervised clinic-based setting often does not change [7–10].

Daily activity levels are comprised of time spent sedentary (i.e. sitting, reclining or lying with low energy expenditure [11]), standing and in other light intensity activities (including incidental movements), and moderate-to-vigorous intensity physical activities (MVPA) [12]. Population estimates of physical activity report that 60% of adults spend < 30 min per day in MVPA [13]. Therefore, for most adults the majority of their waking day is spent in non-MVPA activities. There is now considerable evidence that the distribution of time spent between these non-MVPA activities has a significant impact on health, with time accrued in prolonged, unbroken bouts of sitting potentially particularly harmful (e.g. poor cardio-metabolic health markers and musculoskeletal fatigue and pain) [14–17]. Evidence to this effect has prompted the inclusion of recommendations for sedentary behavior in physical activity guidelines [18]. Moreover, these data suggest that a whole-of-day approach to physical activity promotion should also be considered, where changes in activities across the intensity spectrum are targeted with the focus placed on the time and the context in which activities are accrued [19, 20]. With this approach, the emphasis has moved away from just increasing time in MVPA to also reallocating time away from sedentary to non-sedentary activities (including light and MVPA intensities). However, this whole-of-day approach is yet to be applied in an exercise rehabilitation setting, or to people with cancer or survivors. Their high levels of sedentary time compared to their non-cancer survivor counterparts [21], and increased risk of comorbid chronic disease [22] mean people with cancer and survivors are particularly likely to benefit from this whole-of-day approach.

mHealth (the use of mobile technology to deliver health-related programs) is well suited to supporting this whole-of-day approach to activity promotion. One highly researched and effective mode of mHealth for physical activity promotion is mobile telephone text messages [23–27]. Text messages can: prompt behaviors in real time across the whole day; efficiently deliver tailored, repeated contacts; be delivered to participants to maintain contact over long periods of time; and, maintain two-way communication with an interventionist using minimal resources. Physical activity interventions have previously been delivered successfully via text messages [26–28], including in the context of leveraging off more intensive initial contact [29].

Two previous studies have investigated the efficacy of text messages in cancer survivors for increasing and/or maintaining MVPA [30, 31]. Spark and colleagues used text messaging to target maintenance of MVPA, as well as weight loss and dietary behaviors, following a 6-month telephone delivered weight loss intervention [30], while Gell and colleagues used text messaging in conjunction with health coaching and consumer-based activity trackers to try to enhance MVPA levels [31]. While these studies demonstrated potential for the efficacy of text messaging, both studies were limited by single group, pre-post designs and targeted primarily participation in MVPA, rather than addressing activity across the spectrum. Further, neither study considered the context of the changes in MVPA, focusing on average change in MVPA alone. Including appropriate measurement tools that allow for exploration of when the changes occurred, or the type of behaviors that were being modified by the intervention can increase understanding of intervention effects and can highlight opportunities for further intervention.

Therefore, the aim of this randomized controlled trial was to evaluate the feasibility, acceptability and efficacy of a text message-enhanced clinical exercise rehabilitation intervention using a whole-of-day approach to reduce sitting time and increase activity in people living with and beyond cancer. In addition to the magnitude of change in sedentary behavior and physical activity, the context of these changes were also examined using a time-use approach.

## Methods

### Study design

This randomized controlled trial evaluated a text message-enhanced clinical exercise rehabilitation program compared to the standard clinical exercise rehabilitation program alone. This trial commenced recruitment in February 2016 and data collection was completed in July 2016. This study received ethical clearance from a Medical Research Ethics Committee at The University of Queensland (protocol number: HMS12/1804).

### Participant recruitment

Participants were recruited for this study from the 2016 Clinical Exercise Rehabilitation Program for People with Cancer and Survivors at the School of Human Movement and Nutrition Sciences at The University of Queensland. The target sample size of this study was limited by the capacity of the clinic; the program was able to recruit a maximum of 40 participants (primarily limited by staffing and space requirements). All participants enrolled in the program were invited to participate in the current study; prior to the commencement of the program they were forwarded a Participant Information Sheet and Consent Form via mail or email and then contacted by telephone to establish their eligibility and answer any questions. Eligibility criteria included men and women aged > 18 years of age with a previous histologically-confirmed diagnosis of cancer (excluding childhood cancers), who were at least one-month post-surgery and owned a mobile phone. There were no inclusion criteria with respect to primary cancer diagnosis, or time since diagnosis or treatment. All eligible participants were required to have approval to participate from their medical practitioner as well as provide relevant information (e.g. comorbidities, pre-existing injuries, medications) to assist with exercise testing and prescription. Participants were excluded if they had cardiopulmonary or metabolic disorders that would have prevented safe participation in the testing or exercise sessions. Written informed consent was received from all participants prior to being formally included in the study.

### Randomization

Once informed consent was obtained, participants underwent baseline assessments and were then randomized to participate in the text message-enhanced clinical exercise rehabilitation program (enhanced clinic) or the clinical exercise rehabilitation program alone (standard clinic). Randomization was conducted using an online tool (www.randomization.com) and allocation was carried out by a trained researcher with no involvement in the current study. As the clinical exercise rehabilitation program was delivered via four groups per week (approximately 10 people per group), participants were block randomized to ensure equal distribution of standard- and enhanced- clinic participants.

### Measures

Device-based and self-report activity outcomes, and time spent in self-reported domains of time use were collected at baseline (prior to commencement of the clinic), 4-weeks (immediately after completion of the standard program) and 12-weeks (at completion of the enhanced program). Prior to baseline testing, participant demographic characteristics were determined from the medical history form provided by participants’ medical practitioners. Variables collected included age (years), gender, primary cancer diagnosis, time since diagnosis (months), time since treatment cessation (months) and the number of current medications.

### Feasibility and acceptability outcomes

During the clinical exercise rehabilitation program, the supervising Accredited Exercise Physiologist (AEP) recorded session attendance, adverse (i.e. any untoward medical events occurring during the exercise sessions), and serious adverse (i.e. any adverse events requiring medical attention) events. For the text message-enhanced program, the implementation of each intervention component was also monitored through manual recording by the health professional delivering the session (e.g. duration of face-to-face tailoring sessions), or via the automated software platform used to deliver the text messages (e.g. number of text messages sent and received per participant). Participant’s perceived usefulness and satisfaction with the text messages was evaluated at the 12-week assessment via a 5-point Likert scale (1 = not at all satisfied/useful; 5 = extremely satisfied/useful).

### Efficacy outcomes

#### Device-based activity outcomes

Device-based activity outcomes examined were time spent: sitting, prolonged sitting (sitting for ≥30 min continuously), standing, stepping at a light [< 3 Metabolic Equivalents (METs)] and moderate-to-vigorous (≥3 METs) intensity, as well as usual sitting bout duration (× 50%). Lower values of × 50% are desirable as they indicate a tendency to accrue sitting in shorter rather than longer bouts [32]. These activity outcomes were measured at baseline and at the 4-week and 12-week follow-up using the activPAL micro (PAL Technologies Ltd., Glasgow, United Kingdom). The activPAL micro is a small lightweight triaxial accelerometer that provides accurate measures of sitting, standing, stepping and postural transitions [33–35]. The acceleration data records (as summed vector magnitudes over a 15-s time window) can be used to estimate METs with acceptable validity that is consistent with other devices [36].

Details of procedures for fitting the monitor, initialising and downloading the device, as well as data reduction are outlined in detail in Additional file 1: Table S1. The procedures are consistent with common practice in the field [37]. Briefly, the protocol asked participants to wear the device 24 h/d for seven continuous days, secured by trained staff to the right thigh via medical adhesive, while reporting sleep, wake and removals longer than 10 min in a diary. Based on the diary, non-wear time, sleep, and invalid days (< 10 h waking wear time, ≥95% of time spent in any one activity, or < 500 strides) were excluded. Participants with ≥1 day of valid data were included. Time spent in the various activities were all reported standardized to time awake wearing the monitor (min/16-h awake).

#### Self-report activity outcomes and domains of time use

Sedentary behaviour, MVPA and use of time were measured using the adult version of the Multimedia Activity Recall for Children and Adults (MARCA) at baseline, 4-weeks and 12-weeks. The MARCA is a valid and reliable self-report recall instrument with test-retest intraclass correlation coefficients of 0.99–1.00 for a number of key outcomes (including MVPA, screen time and sleep) [38] and convergent validity of rho = 0.72 for MVPA compared to accelerometry [38] and r = 0.77 for sedentary behaviour compared with activPAL [39]. Participants were asked to recall their previous 24 h from midnight to midnight in time slices as small as 5 min, anchored by meal times throughout the day (breakfast, lunch and dinner) and in the sequence in which they occurred. Administered by computer-assisted telephone interview, the interviewer codes each reported activity by choosing from over 500 discrete activities organized under categories. The activities are then linked to a compendium of energy costs in METs, largely based on the Compendium of Physical Activities [40]. In addition, each activity has a unique code indicating body posture (lying, sitting, standing, in locomotion) [39] and is categorised into one of nine ‘super-domains’ of time use: Sleep, Chores, Cultural, Physical Activity, Quiet Time, Screen Time, Self-Care, Social and Work and Study [19]. At each time point, two separate calls were made 1 week apart, during which participants recalled the two previous days. At each time point, participants therefore recalled 4 days of activity, including at least one weekday and one weekend day. Daily time in the outcomes of interest were then determined by summing the time spent in each activity per day, then averaging across the recalled days using a 5:2 weighting for weekdays:weekend days to capture typical activity patterns [19].

Total sedentary time was determined by calculating the time spent in waking activities with either a sitting or lying body position code. MVPA was determined by calculating the time spent in activities with an intensity of ≥3 METs. Use of time was determined by calculating the time (min/d) spent in each of the nine MARCA super-domains of time use. The most frequently reported activities (minutes) were determined by calculating the total duration of time spent in each activity in the compendium for each intervention group, at each time point.

### Interventions

#### Standard clinic

All study participants completed the standard four-week clinical exercise rehabilitation program designed and delivered specifically for people with cancer and survivors. The program consisted of four, 1-h individualized exercise sessions over 4 weeks, supervised by an AEP, with supplementary home-exercise programs detailing prescribed exercises. The specific exercise prescription and design of the program has been described previously [7], and was found to be safe, feasible and efficacious in improving physical and psychosocial health in people with cancer and survivors.

#### Enhanced clinic

In addition to the four-week clinical exercise rehabilitation program, participants randomly allocated to the enhanced clinic also received 12-weeks of tailored text messaging. The text messaging was designed to improve whole-of-day activity by targeting: reducing overall sedentary time; breaking up prolonged, unbroken periods (30+ mins) of sedentary time with light intensity activities (including standing); and, increasing MVPA behavior (in addition to the MVPA performed during the clinic sessions). The text messages were individually-tailored for frequency, timing, content and wording. Data to tailor the texts were collected during two sessions (tailoring sessions) prior to, and following completion of, the four-week clinical exercise rehabilitation program.

##### Baseline tailoring session

The face-to-face tailoring sessions were delivered by a ‘coach’, an allied health professional (Physiotherapist or AEP), using a guided script. Each session took approximately 20–30 min. The coaches received a 2 h, study-specific training session and an accompanying 13-page training manual. The baseline tailoring session served to: introduce participants to a whole-of-day approach to activity; provide brief education on the health benefits of reducing time spent sitting and increasing physical activity; build rapport between the participant and coach; and, gather information to tailor the text message content, frequency and timing for the first 4 weeks of the program. During the session, participants were asked to reflect on their activity patterns to identify periods of prolonged sitting (i.e., sitting danger zones) and opportunities for physical activity. To guide this reflection, the coach showed the participant their sitting and physical activity patterns using heat maps (visual display of 24 h body posture and intensity; see example in Additional file 2: Figure S2) from activPAL data; and, temporograms (visual display of 24 h time use; see example in Additional file 3: Figure S3) from the MARCA completed during the baseline assessment. Participant data collected during this tailoring session [i.e., participant name, coach name, sitting danger zones (× 2), strategies to break sitting danger zones (× 2), MVPA goal, MVPA perceived barriers to goal (× 2), MVPA solutions to barriers (× 2)] were entered in a purpose-designed software platform that enables users to create, send and track personally-tailored text messages (*Propelo™*, www.propelo.com.au).

##### Text messages

The physical activity text messages in this program were modified from messages previously shown to be effective at supporting MVPA changes in breast cancer survivors and a general adult population [30, 41]. Further modifications were made to the tone and language of the text messages for this population (i.e. no abbreviations, avoided using the terms ‘slip’ or ‘fall behind’ in relation to behavioral relapses). Specific sedentary behavior text messages were developed, given the behavior change techniques targeting MVPA are not always applicable to sedentary behavior [42]. The pervasive and habitual nature of sedentary behavior means that it is well suited to real-time prompting to raise contextual awareness and prompt action. Education on the potential health consequences of sitting and how patterns of sitting time are accrued was considered a key element. Therefore, the text messages in this trial targeted: 1) education (for sedentary behavior and MVPA); 2) real-time behavioral prompting (for sedentary behavior and MVPA); and, 3) goal checking (for MVPA; See Table 1 for examples).

**Table 1:** Type and frequency of text messages sent to the enhanced clinic group

Text message types (targeted behavior)	Range of Frequency	Example Text
Educational tips (SB and MVPA)	Fixed: 2 per fortnight	Bob. Just standing up & stretching your legs can help your heart! Stand up today. Kylie
Real-time behavioral prompts (SB)	Variable: 2–10 per fortnight	Hi Bob. You’re probably watching TV now. Enjoy the downtime - but try to stand up & move in every ad break. Kylie
Real-time behavioral prompts (MVPA)	Variable: 1–4 per fortnight	Bob you wanted to walk early in the morning. Put your shoes out beside your bed now as a reminder. Kylie
Goal checks (MVPA)	Fixed: 1 per fortnight	Hi Bob. Did you reach your goal to walk 4 x 40mins this week? Text me back yes or no. Kylie
Goal check replies (MVPA)	Dependent on participant reply	We all lose motivation at times Bob. I know you can do it this week. Focus on your desire to be healthy - I am here to help you! Kylie

*SB* sedentary behavior, *MVPA* moderate-to-vigorous physical activity

The frequency of the text messages sent varied among participants depending on personal preference, with a minimum of six text messages per fortnight for the duration of the 12-week intervention. Text messages included a minimum of two educational tips, three real-time prompts and one goal check text per fortnight (see Table 1). All text messages were signed off using the first name of the coach who delivered the baseline tailoring session. Text messages were generated and sent by research staff using the *Propelo™* platform, which enabled messages to be tailored and pre-programmed in advance and scheduled to be sent at specific times to encourage real-time behavioral prompting (e.g. Wednesday afternoon at 3:05 pm).

Replies to the goal check text were stored and a tailored response was automatically triggered if the participant replied with the words ‘yes’ or ‘no’ (or accepted variations of these, e.g. yep). If participants replied to a goal check text with additional words (e.g. providing justification as to why they did not reach their weekly goal) or a word not identified as a variant of ‘yes’ or ‘no’, then the program sent an email to research staff. Research staff then manually selected which tailored goal check reply (met goal or did not meet goal) was to be sent to the participant. If required, the research staff contacted the participant’s coach to provide tailored advice depending on what information the participant supplied in their text.

##### Follow-up tailoring session

At the completion of the four-week supervised clinical rehabilitation program, participants received a second follow-up, face-to-face tailoring session to update their preferences for text message frequency, timing and content for the remaining 8 weeks of the program. The session was led by the same coach as the baseline tailoring session. The follow-up tailoring session was designed to last approximately 20 min and was guided by a script.

### Statistical analysis

Statistical analyses were performed in STATA version 14 (StataCorp LLC [US]) with significance set to *p* < 0.05, two tailed. Participant characteristics, process outcomes and adverse outcomes were reported using descriptive statistics. Mixed models were used to examine change in activity, with outcomes examined as change scores (follow-up- baseline). Models included effects for time (4-weeks/12-weeks), group (clinic/enhanced clinic), group x time interaction (12-weeks-4 weeks), baseline values of the outcome variable (to control regression to the mean) [43] and other potential confounders: age (years); gender (male/female); time since diagnosis (months); and, number of medications currently taken (0 / 1 / ≥2). From the model, we report marginal means, and comparisons of marginal means. Consistent with intention to treat principles, participants were analysed as randomized. Analyses were of evaluable cases, with all participants (100%) followed up at 4 weeks and 86% (*n* = 31) followed up at 12 weeks.

The primary aim of this study was to assess the feasibility and acceptability of the protocol, and to provide estimates of the effect on daily sitting time (16 h waking day) as the primary outcome. The sample size of the study was limited by the capacity of the exercise rehabilitation program (40 participants). However, allowing for an 80% uptake and 10% drop out [7] (*n* = 28; *n* = 14 per group), the study was adequately powered to detect a medium effect (Cohen’s d = 0.5) between groups, with 80% power and 5% alpha.

## Results

### Participants

**Fig. 1:**
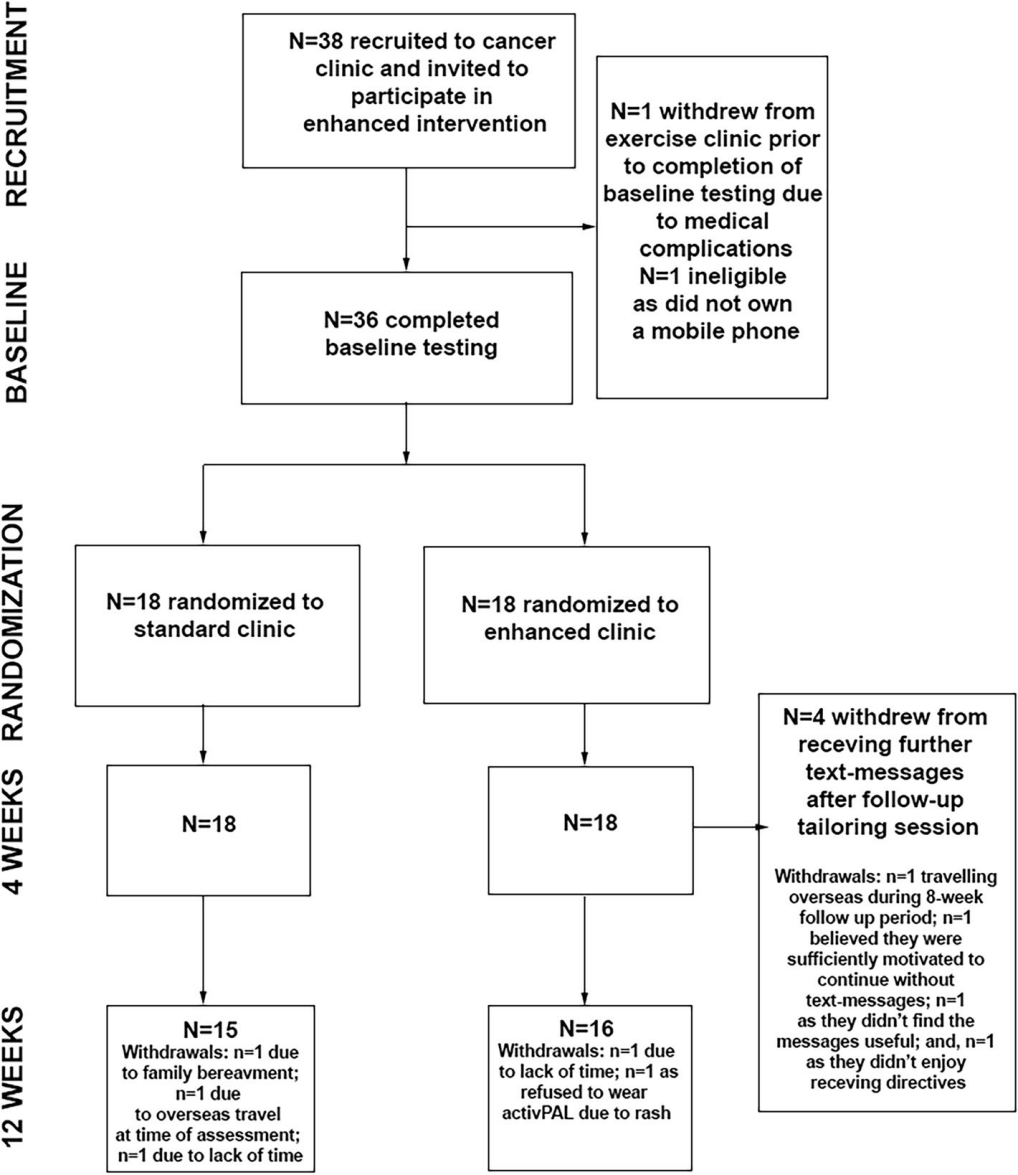
Participant flow diagram

Thirty-eight participants were recruited (Fig. 1), with 36 completing baseline testing and being randomly allocated to either the standard or enhanced clinic groups (*n* = 18, respectively). One participant was excluded due to eligibility criteria (they did not own a mobile phone) and the other was unable to complete baseline testing due to medical complications. Participant demographics are described in Table 2. Participants were, on average, approximately 65 years old, slightly overweight, mostly female and taking two or more medications. Most participants had a primary diagnosis of colorectal cancer. Four (10%) participants were currently undergoing treatment, with an average time since treatment of four years over the whole sample.

**Table 2:** Participant baseline characteristics

	Standard clinic (*n* = 18)	Text message-enhanced clinic (n = 18)	Overall (*n* = 36)
Age (years)	61.3 ± 9.1	68.3 ± 9.1	64.8 ± 9.6
Body mass index (kg/m^2^)	26.1 ± 4.2	26.5 ± 3.9	26.3 ± 4.0
Gender (male)	11 (61%)	12 (67%)	23 (64%)
Primary cancer			
Colorectal	12 (67%)	13 (72%)	25 (69%)
Prostate	5 (28%)	5 (28%)	10 (28%)
Breast	1 (6%)	0 (0%)	1 (3%)
Number of medications			
None	4 (22%)	4 (22%)	8 (22%)
1	3 (17%)	2 (11%)	5 (14%)
≥ 2	11 (61%)	12 (67%)	23 (64%)
Post-diagnosis (months)	54.5 (38.0, 72.0)	60.0 (47.0, 71.0)	57.5 (43.5, 71.5)
Post treatment (months)	38.5 (25.0, 66.0)	51.0 (10.0, 61.0)	46.0 (24.5, 61.0)
Activity (activPAL micro)^a^			
Wear time (h)	15.7 ± 0.7	15.9 ± 0.9	15.8 ± 0.8
Sitting (h/16 h)	9.4 ± 2.1	9.5 ± 1.8	9.4 ± 1.9
Prolonged sitting (h/16 h)	4.6 ± 2.2	4.7 ± 1.8	4.7 ± 2.0
Standing (h/16 h)	4.3 ± 1.5	4.3 ± 1.3	4.3 ± 1.4
Stepping (h/16 h)	2.4 ± 0.8	2.1 ± 0.7	2.3 ± 0.8
Light stepping (min/16 h)	95.3 ± 26.3	98.2 ± 22.8	96.7 ± 24.3
MVPA (min/16 h)	43.7 ± 28.8	31.1 ± 19.4	37.4 ± 25

*MVPA* moderate-to-vigorous physical activity. Table presents n (%), mean ± standard deviation or median (25th, 75th percentile) as appropriate

^a^Average daily activity monitored on valid days (≥10 h wear, ≥500 strides, < 95% of time in any one activity), excluding non-wear and sleep

All participants were retained at 4-week follow-up, while five participants withdrew (*n* = 3 standard clinic; *n* = 2 enhanced clinic) between 4- and 12-weeks, namely due to lack of time, family bereavement, adverse reaction to activPAL dressings and overseas travel (Fig. 1). Missing data at 12 weeks did not differ significantly (*p* = 0.603) between the enhanced clinic (6%; *n* = 1) and standard clinic (17%; n = 3) groups.

### Feasibility and acceptability

Thirty-one (out of 36) participants (86%) attended all four supervised exercise sessions. Overall, out of 144 total sessions, attendance was 96% (138/144). One adverse event was recorded; a participant overbalanced and fell during a lunging exercise. No injury occurred and no follow-up treatment was required. All 18 participants in the enhanced clinic group completed both the baseline and follow-up tailoring sessions, and received text messages during the first 4 weeks of the program (whilst attending the clinical exercise rehabilitation program); however, four participants opted out of receiving text messages for the remaining 8 weeks. Reasons for opting out of the texts after the first 4 weeks included: travelling overseas during the 8-week follow-up period (*n* = 1); sufficiently self-motivated to continue without texts (n = 1); not finding the texts useful (n = 1); and, not liking the directive language in the texts (n = 1). On average, participants opted to receive eight text messages per fortnight (range = 6–12 text messages/fortnight, possible range: 6–17 per fortnight). During the baseline tailoring session, 13/18 opted to receive the minimum of two prompts per fortnight for sedentary behavior and 12/18 opted for the minimum of one prompt per fortnight for the MVPA. The average reply rate to the fortnightly MVPA goal checks was 78%, with 8/18 participants replying to all of the goal checks they received. Of the 83 goal check replies received from participants, eight (9.6%) were screened by the coach for appropriateness of content before sending a reply.

During the initial tailoring session, each participant nominated two ‘sitting danger zones’ to target. The most commonly targeted zones identified were: watching television (12/36 danger zones); computer work (11/36 danger zones); and, reading (8/36 danger zones). Each participant also set an MVPA goal during the initial tailoring session (which may have included more than one type of activity). Here, the most commonly nominated activities were brisk walking (12/18 goals); cycling (5/18 goals); and, going to the gym (4/18 goals).

Seventeen participants in the enhanced clinic completed the satisfaction survey; results are shown in Table 3. Mean (±SD) satisfaction scores were high overall (4.3 ± 0.8) and for both the tailoring sessions and the tailored text messages. Most participants read the text messages, had no difficulty understanding them, and approximately half found the program elements extremely helpful in meeting their physical activity and sitting goals. Of the 14 participants who responded to a request for program suggestions: nine commended the program without raising any suggestion or complaint; two indicated that the texts had not succeeded in prompting a sense of motivation (sometimes prompting guilt); one participant indicated the texts content was incongruent with what was happening when it was received; one participant wanted more texts that required a response; and, one participant suggested access to online exercise prescription.

**Table 3:** Satisfaction and usefulness of the enhanced clinic intervention (*n* = 17)

	Tailoring Sessions	Text Messages
Satisfaction score (1–5), *mean ± SD*	4.5 ± 0.6	4.1 ± 1.1
Extremely satisfied, *n(%)*	12 (71%)	9 (53%)
Extremely useful for meeting physical activity goal, n(%)	10 (59%)	8 (47%)
Extremely useful for meeting sitting goal, n(%)	8 (47%)	8 (47%)
Read the text messages, n(%)^a^	–	16 (100%)
Ease of understanding text messages ^a^		
Difficult/ very difficult	–	0 (0%)
Easy	–	2 (12%)
Very easy	–	14 (88%)

^a^ excludes n = 1 participant with missing data

### Efficacy

#### Device-based activity outcomes

Changes in activPAL-assessed activity from baseline to 4-weeks, baseline to 12-weeks and 4-weeks to 12-weeks for both the standard and enhanced clinic groups are presented in Table 4. Over the first 4 weeks, none of the objectively-measured activities outside the clinic changed over time or differed between groups to a large or statistically significant degree. However, at 12-week follow-up, the enhanced clinic group reduced their overall sitting and prolonged sitting time by 40–50 min/16 h awake, with corresponding increases in standing and light-intensity stepping. The standard clinic group made no large or significant changes in objectively measured activities at 12 weeks or between 4- and 12-weeks. Differences between groups reached statistical significance for total sitting and light-intensity stepping, with non-significant tendencies towards better outcomes in the enhanced clinic group than the standard group in prolonged sitting, standing and sitting bout duration. The size of the differences between the 4-week and 12-week assessments indicated that most of the improvements in the enhanced clinic group occurred over this timeframe, with significant improvements seen for overall and prolonged sitting time, standing time, and light-intensity stepping. None of the changes or group differences in activPAL-assessed MVPA observed were statistically significant or large; however, some amounted to half an hour per week or more and confidence intervals could not rule out effects that were of a meaningful magnitude consistent with 1-h or more per week.

**Table 4:** Changes in out-of-clinic activity (activPAL; min/16 h awake) in the standard clinic (C) and text message-enhanced clinic (EC)

		Week 4 (clinic cessation)			Week 12 (text cessation)			Week 12-Week 4	
		n	M (95%CI)	p	n	M (95%CI)	p	M (95%CI)	p
Sitting (min/16 h awake)	C	18	1.7 (−18.4, 21.8)	0.869	15	−0.5 (−30.2, 29.3)	0.975	−2.2 (−29.9, 25.6)	0.878
	EC	18	−14.7 (−34.8, 5.3)	0.151	17	**−48.2 (−76.4, − 20.0)**	**0.001**	**−33.5 (−59.7, − 7.3)**	**0.012**
	EC-C		−16.4 (− 46.2, 13.4)	0.281		**−47.7 (−89.9, − 5.6)**	**0.023**	*− 31.3 (−69.5, 6.8)*	*0.107*
Prolonged sitting in ≥20 min bouts (min/16 h awake)	C	18	−8.2 (− 29.7, 13.2)	0.452	15	−8.3 (− 36.7, 20.2)	0.569	0.0 (− 24.8, 24.7)	0.998
	EC	18	−16.3 (−37.7, 5.2)	0.137	17	**−40.6 (−67.8, − 13.5)**	**0.003**	**−24.4 (− 47.7, − 1.1)**	**0.041**
	EC-C		−8.0 (−39.9, 23.8)	0.622		−32.4 (−73.0, 8.3)	0.119	−24.4 (−58.4, 9.6)	0.160
Usual sitting bout duration (min)	C	18	−1.6 (−3.7, 0.5)	0.143	15	−0.1 (−2.6, 2.3)	0.910	1.4 (−1.0, 3.9)	0.247
	EC	18	−0.7 (−2.8, 1.4)	0.526	17	**−2.8 (−5.1, − 0.4)**	**0.019**	*−2.1 (−4.4, 0.2)*	*0.076*
	EC-C		0.9 (−2.2, 4.0)	0.573		−2.6 (−6.2, 0.9)	0.143	**−3.5 (− 6.9, −0.2)**	**0.039**
Standing (min/16 h awake)	C	18	−4.4 (−18.4, 9.5)	0.535	15	2.2 (−30.8, 35.3)	0.894	6.7 (−26.4, 39.7)	0.693
	EC	18	10.4 (−3.5, 24.4)	0.143	17	**44.3 (13.2, 75.4)**	**0.005**	**33.9 (2.8, 65.0)**	**0.033**
	EC-C		14.8 (−6.1, 35.8)	0.164		*42.1 (−3.9, 88.1)*	*0.073*	27.2 (−18.1, 72.6)	0.239
Stepping (min/16 h awake)	C	18	3.7 (−6.8, 14.3)	0.489	15	−0.5 (−14.1, 13.0)	0.940	−4.2 (−17.8, 9.3)	0.538
	EC	18	3.5 (−6.9, 14.0)	0.510	17	2.5 (−10.3, 15.4)	0.698	−1.0 (− 13.8, 11.8)	0.881
	EC-C		−0.2 (−15.7, 15.3)	0.979		3.1 (−16.2, 22.3)	0.756	3.3 (−15.3, 21.9)	0.731
Light stepping (min/16 h awake)	C	18	1.0 (−0.7, 2.7)	0.247	15	0.8 (−4.0, 5.6)	0.749	−0.2 (− 4.6, 4.2)	0.916
	EC	18	1.1 (−0.6, 2.9)	0.191	17	**7.8 (3.2, 12.3)**	**0.001**	**6.6 (2.5, 10.7)**	**0.002**
	EC-C		0.1 (−2.4, 2.7)	0.921		**7.0 (0.4, 13.6)**	**0.039**	**6.9 (0.8, 12.9)**	**0.026**
Moderate-vigorous stepping (min/16 h awake)	C	18	6.2 (−1.9, 14.3)	0.131	15	−0.4 (−7.3, 6.4)	0.906	−6.6 (− 14.7, 1.4)	0.105
	EC	18	1.6 (− 6.3, 9.6)	0.685	17	−3.1 (−9.7, 3.4)	0.345	−4.8 (−12.5, 2.9)	0.222
	EC-C		−4.6 (−16.4, 7.2)	0.447		−2.7 (−12.7, 7.3)	0.593	1.9 (− 9.2, 13.0)	0.743

Table presents mean change over time or mean difference between groups (M) with 95% Confidence Interval (CI) as estimated by mixed models adjusting for baseline values of the outcome, age (years), gender (male/female), time since diagnosis (months) and number of medications currently taken (0/1/≥2). **Bold** text indicates changes that were significant at *p* < 0.05

#### Self-report activity outcomes

Changes in activity according to the MARCA from baseline to 4-weeks, baseline to 12-weeks and from 4- to 12-weeks are presented for both standard and enhanced clinic groups in Table 5. In contrast to the activPAL results, over the first 4 weeks, there were significant differences between groups, with the enhanced clinic group reporting less sleep, more vigorous physical activity, and more MVPA than the standard clinic group. Other outcomes did not differ significantly between the groups. These between-group differences were driven by the standard clinic having significantly changed their reported daily sleep, MVPA and moderate activity while the enhanced clinic group made no significant changes in these reported behaviors and experienced a significant increase in self-report vigorous physical activity.

**Table 5:** Changes in out-of-clinic time use by intensity (MARCA; min/day) in the standard clinic (C) and text message-enhanced clinic (EC)

		Week 4 (clinic cessation)			Week 12 (text cessation)			Week 12-Week 4	
		n	M (95%CI)	p	n	M (95%CI)	p	M (95%CI)	p
Sleep	C	17	**51.2 (18.1, 84.4)**	**0.002**	15	21.3 (−13.6, 56.2)	0.232	*−29.9 (−61.2, 1.4)*	*0.061*
	EC	18	1.9 (−30.1, 33.8)	0.909	15	**36.0 (1.5, 70.5)**	**0.041**	**34.1 (3.0, 65.2)**	**0.032**
	EC-C		**−49.3 (−97.7, −1.0)**	**0.046**		14.7 (− 36.7, 66.1)	0.575	**64.0 (19.9, 108.2)**	**0.004**
Sitting	C	17	−10.4 (−54.8, 34.1)	0.648	15	14.4 (−37.7, 66.6)	0.587	24.8 (−18.4, 68)	0.260
	EC	18	−29.5 (−72.5, 13.5)	0.178	15	**−65.7 (−117.2, − 14.2)**	**0.012**	− 36.2 (− 79.2, 6.9)	0.100
	EC-C		−19.2 (−84.5, 46.2)	0.566		**−80.1 (− 156.5, − 3.8)**	**0.040**	*− 61.0 (− 121.9, 0.0)*	*0.050*
LPA	C	17	2.1 (−31.9, 36.1)	0.903	15	15.5 (−13.6, 44.6)	0.297	13.4 (−23.8, 50.6)	0.481
	EC	18	13.6 (−19.1, 46.4)	0.415	15	8.4 (−20.7, 37.6)	0.570	−5.2 (−41.8, 31.4)	0.782
	EC-C		11.5 (−37.7, 60.7)	0.647		−7.1 (−50.5, 36.4)	0.750	−18.6 (−70.8, 33.6)	0.486
MPA	C	17	**−37.2 (−72.3, −2.2)**	**0.037**	15	**−52.9 (−80.3, − 25.6)**	**< 0.001**	−15.7 (− 52.7, 21.3)	0.405
	EC	18	−1.8 (− 35.6, 32.0)	0.916	15	−7.5 (− 35.0, 20.0)	0.592	− 5.7 (−42.1, 30.7)	0.759
	EC-C		35.4 (−14.9, 85.7)	0.168		**45.4 (4.4, 86.4)**	**0.030**	10.0 (−41.8, 61.8)	0.705
VPA	C	17	0.2 (−6.3, 6.6)	0.956	15	5.2 (−12.0, 22.4)	0.554	5.0 (−12.4, 22.4)	0.573
	EC	18	**11.4 (5.1, 17.7)**	**< 0.001**	15	**19.6 (2.5, 36.8)**	**0.025**	8.2 (−9.2, 25.6)	0.354
	EC-C		**11.2 (1.5, 20.9)**	**0.023**		14.4 (−10.1, 39.0)	0.249	3.2 (−21.4, 27.8)	0.798
MVPA	C	17	**−40.1 (−75, −5.3)**	**0.024**	15	**−50.1 (−79.1, − 21.1)**	**0.001**	− 10.0 (− 41.5, 21.5)	0.534
	EC	18	13.0 (−20.5, 46.6)	0.446	15	17.1 (−11.7, 46.0)	0.244	4.1 (−27.0, 35.2)	0.797
	EC-C		**53.2 (2.9, 103.5)**	**0.038**		**67.3 (24.0, 110.6)**	**0.002**	14.1 (−30.1, 58.3)	0.533

*LPA* light physical activity, *MPA* moderate physical activity, *VPA* vigorous physical activity, *MVPA* moderate-to-vigorous physical activity. Table presents mean change over time or mean difference between groups (M) with 95% Confidence Interval (CI) as estimated by mixed models adjusting for baseline values of the outcome, age (years), gender (male/female), time since diagnosis (months) and number of medications currently taken (0/1/≥2). **Bold** text indicates changes that were significant at *p* < 0.05

At the 12-week follow-up, the enhanced clinic group reported significantly less sleep and more vigorous and MVPA, than the standard clinic group. These effects occurred due to decreased MVPA and moderate physical activity by 50–53 min/day in the standard clinic group, while the enhanced clinic group decreased their reported sitting time by approximately 1 hour per day and increased their vigorous activity and sleep by 20 and 40 min per day, respectively.

#### Self-report time use domain outcomes

Changes in time use domains according to the MARCA from baseline to 4-weeks, baseline to 12-weeks and from 4- to 12-week follow-up for both the standard and enhanced clinic groups are presented in Table 6. There were no significant differences between the groups in the change in time spent in the use of time domains over the first 4 weeks of the intervention. Both groups either significantly increased time spent in Physical Activity or tended to do so (+ 8–9 min/day). The standard-clinic group significantly decreased their Social time by 40 min/day, whilst there was no significant change in the enhanced clinic group. Difference between the groups were apparent at 12 weeks, however, with the enhanced clinic group reporting significantly lower (by approximately 1 h/day) Screen Time and Quiet Time than the standard-clinic group at 12-weeks. Both groups, to a similar degree, significantly reduced their time spent in cultural activities (− 5 min/day) and increased their time spent in Physical Activity (+ 15–17 min/day). Other differences between groups and changes were not statistically significant, though some were of a meaningful magnitude. For example, the enhanced clinic group spent approximately half an hour per day more in Work and Study than the standard-clinic group at 12 weeks.

**Table 6:** Changes in out-of-clinic time use by domains (MARCA; min/day) in the standard clinic (C) and the text message-enhanced clinic (EC)

		Week 4 (clinic cessation)			Week 12 (text cessation)			Week 12-Week 4	
		n	M (95%CI)	P	n	M (95%CI)	P	M (95%CI)	p
Chores (min/day)	C	17	−19.2 (−51.9, 13.5)	0.249	15	−26.3 (−64.5, 11.9)	0.177	−7.1 (−47.7, 33.6)	0.734
	EC	18	−21.5 (−53.0, 10.1)	0.182	15	−11.1 (−49.2, 27.0)	0.568	10.4 (−29.9, 50.7)	0.614
	EC-C		−2.2 (−50.1, 45.7)	0.928		15.2 (−41.0, 71.4)	0.5962	17.4 (−39.8, 74.6)	0.550
Cultural	C	17	−2.0 (−11.6, 7.6)	0.682	15	**−4.7 (−6.6, − 2.9)**	**< 0.001**	− 2.7 (− 13.2, 7.8)	0.612
	EC	18	−0.2 (−9.5, 9.1)	0.964	15	**−4.7 (−6.6, − 2.9)**	**< 0.001**	− 4.5 (− 14.7, 5.6)	0.382
	EC-C		1.8 (−11.6, 15.2)	0.794		0.0 (−2.8, 2.7)	0.978	−1.8 (− 16.4, 12.8)	0.806
Physical Activity	C	17	8.1 (−0.1, 16.4)	0.054	15	**17.1 (2.7, 31.5)**	**0.020**	9.0 (−1.9, 19.9)	0.107
	EC	18	**9.1 (1.1, 17.1)**	**0.026**	15	**14.8 (0.6, 29.0)**	**0.041**	5.8 (−5.2, 16.7)	0.301
	EC-C		0.9 (−11.3, 13.1)	0.881		−2.3 (−22.9, 18.3)	0.828	−3.2 (−18.6, 12.2)	0.682
Quiet time	C	17	7.2 (−17.8, 32.3)	0.571	15	28.0 (−4.5, 60.4)	0.091	20.7 (−15.1, 56.5)	0.256
	EC	18	−14.7 (−38.9, 9.5)	0.233	15	**−35.3 (−67.7, −2.9)**	**0.033**	−20.6 (−56.1, 14.9)	0.256
	EC-C		−21.9 (−58.4, 14.6)	0.239		**−63.3 (−110.5, − 16.0)**	**0.009**	−41.3 (−91.8, 9.1)	0.108
Screen time	C	17	15.1 (−32.0, 62.1)	0.530	15	1.4 (−56.7, 59.5)	0.962	−13.7 (−61.5, 34.2)	0.576
	EC	18	13.3 (−32.0, 58.7)	0.564	15	−48.6 (−106, 8.8)	0.097	−1.7 (−70.8, 67.3)	0.960
	EC-C		−50.0 (− 134.8, 34.7)	0.247		**−62.0 (− 109.7, − 14.2)**	**0.011**	− 48.3 (− 115.9, 19.3)	0.161
Self-care	C	17	−1.9 (− 18.3, 14.4)	0.818	15	3.8 (−12.1, 19.7)	0.638	5.7 (−12.6, 24.1)	0.541
	EC	18	1.7 (−14.2, 17.5)	0.836	15	0.5 (−15.3, 16.4)	0.949	−1.1 (− 19.3, 17.0)	0.901
	EC-C		3.6 (−20.2, 27.4)	0.768		−3.3 (− 26.9, 20.3)	0.784	−6.9 (− 32.7, 18.9)	0.601
Social	C	17	**−41.0 (−67.9, −14.1)**	**0.003**	15	−22.3 (−62.2, 17.6)	0.274	18.7 (−18.6, 56.0)	0.326
	EC	18	−22.5 (−47.9, 2.8)	0.081	15	0.2 (−39.5, 39.8)	0.994	18.4 (−22.0, 58.8)	0.371
	EC-C		22.4 (−36.5, 81.3)	0.455		22.7 (−14.5, 59.9)	0.232	4.0 (−48.7, 56.7)	0.881
Transport	C	17	4.5 (−23.0, 32.1)	0.747	15	1.2 (−24.4, 26.7)	0.929	−3.4 (−37.9, 31.1)	0.848
	EC	18	−6.4 (−33.1, 20.2)	0.636	15	11.8 (−13.8, 37.4)	0.368	18.2 (−15.8, 52.2)	0.294
	EC-C		−11 (−50.6, 28.6)	0.587		10.6 (−27.0, 48.2)	0.581	21.6 (−26.9, 70.0)	0.383
Work & Study	C	17	−4.9 (−33.3, 23.6)	0.738	15	−3.0 (− 32.8, 26.8)	0.846	1.9 (−23.5, 27.3)	0.883
	EC	18	26.9 (−0.7, 54.5)	0.056	15	27.1 (−2.4, 56.5)	0.072	0.2 (−25.1, 25.4)	0.991
	EC-C		31.8 (−12.3, 75.8)	0.158		30.0 (− 16.1, 76.1)	0.202	− 1.7 (−37.5, 34.0)	0.924

Table presents mean change over time or mean difference between groups (M) with 95% Confidence Interval (CI) as estimated by mixed models adjusting for baseline values of the outcome, age (years), gender (male/female), time since diagnosis (months) and number of medications currently taken (0/1/≥2). **Bold** text indicates changes that were significant at *p* < 0.05

**Fig. 2:**
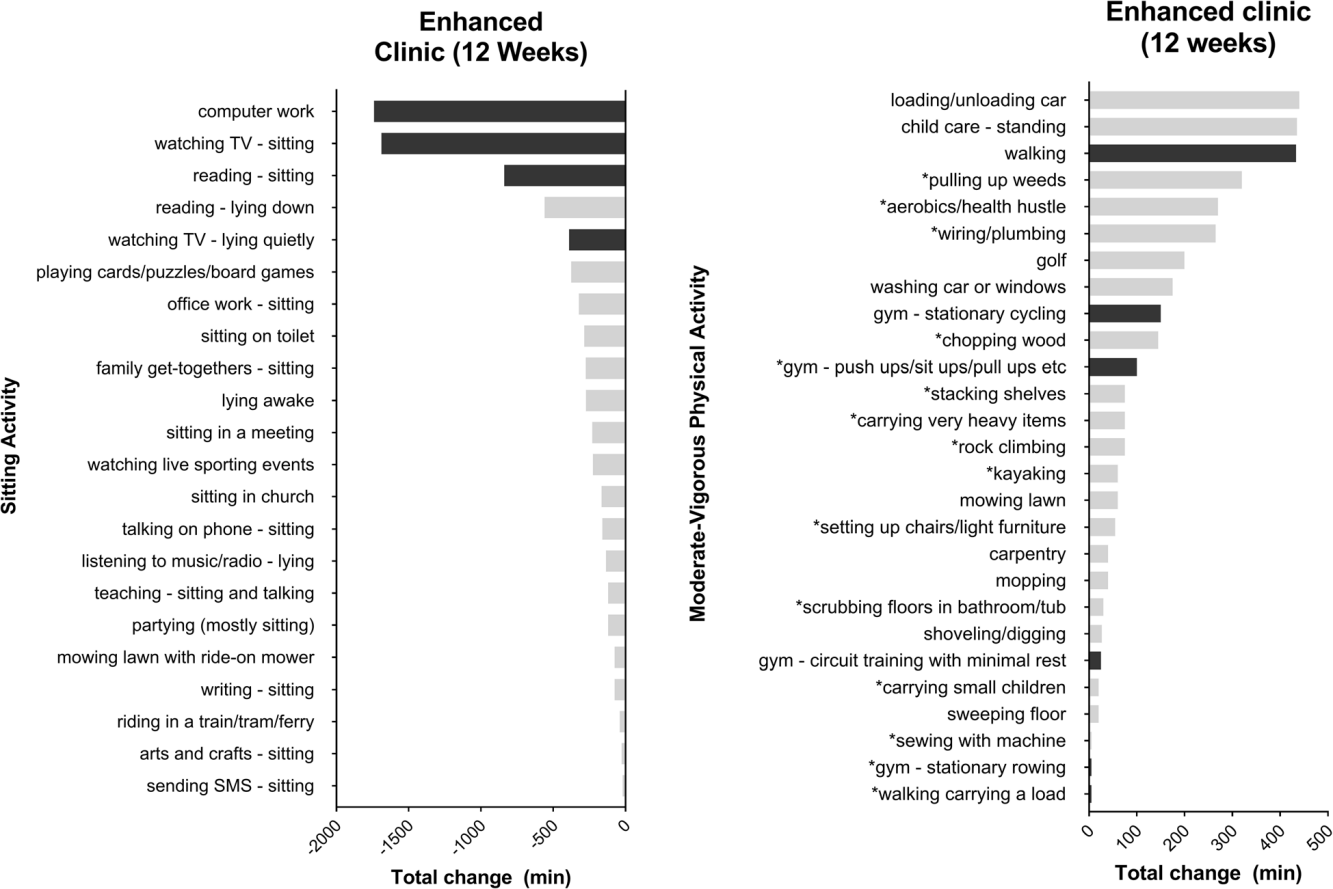
Baseline to 12-week follow-up changes in most frequently reported sedentary behavior and moderate-to-vigorous physical activity activities from the MARCA by duration (min/week) in the text message-enhanced clinic. *Note:* Activities indicated by an asterisk are activities that are reported for the first time at follow-up and those shaded in black are activities that map directly onto commonly chosen intervention targets. MARCA = Multimedia Activity Recall for Children and Adults

Change in self-reported activities according to the MARCA from baseline to 12-week follow up are presented in Fig. 2 for the enhanced clinic group (baseline to 4-weeks presented in Additional file 4: Figure S4). Consistent with intervention targets, participants reported more time spent walking and in gym-related activities as well as reductions in computer work, watching TV and reading.

## Discussion

This study explored the feasibility, acceptability and efficacy of a text message-enhanced clinical exercise rehabilitation program for reducing sitting and increasing activity in people living with and beyond cancer. The major findings of this study were that the enhanced intervention was feasible to deliver, participants reported it to be acceptable and that it resulted in significant reductions in sitting (approximately 1 h/day) and increases in MVPA (approximately 1 h/day) compared to the standard clinic group. This was predominantly due to activity changes after completion of the supervised exercise phase. This study also demonstrated that the specific targets chosen by participants (i.e. their sitting danger zones and MVPA goals), were positively impacted by the enhanced intervention as shown in the MARCA time use domains data.

The changes in MVPA outcomes in the current trial exceed previously reported changes using other digital health interventions in cancer survivors. A recent meta-analysis found that the average increase in physical activity among cancer survivors following a digital health intervention (e.g. websites, mobile applications or text messaging) was 41 min/week (95% CI 12, 71 min) [44]. While there are no systematic reviews of sedentary behavior interventions in cancer survivors, a meta-analysis of general sedentary behavior interventions in otherwise healthy populations estimated a significant reduction in sitting time of 42 min/day (95%CI -78.92, − 4.60) favoring the intervention, with no significant effect for combined physical activity and sedentary behavior interventions for reducing sedentary behavior [45]. Therefore, findings from the current study demonstrate novel evidence for the efficacy of interventions employing a whole-of-day approach, and further, that simultaneous changes in both sitting and physical activity are feasible, and highly acceptable to participants.

The largest changes in both sedentary behavior and physical activity were observed at 12-weeks. This suggests the potential of the enhanced clinic approach to leverage off existing face-to-face, supervised exercise interventions to support participants’ transition to unsupervised, self-directed behaviors. Importantly, these favorable changes in activity were achieved using an intervention with a demonstrated capacity to be delivered at scale [41]. Scalability is important given the growing number of cancer survivors [1] and the challenge of maintaining behavior change following clinical rehabilitation [46]. Nonetheless, while the current study demonstrated maintenance of behavior change with the text message-enhanced intervention beyond the supervised phase, future studies should assess whether changes are maintained once the text message component has also been withdrawn.

A novel feature of this study, compared to other trials in this field, was the use of the MARCA. The MARCA, which was well received among participants, is a high-resolution, 24 h recall tool that collects activity data in 5 min increments. In the context of this study, the MARCA was used as an intervention resource to assist participants in identifying sitting danger zones and for guiding MVPA goal setting. In addition, the MARCA was used as an outcome measure, allowing an in-depth understanding of the context of the changes in sedentary behavior and physical activity that would have otherwise been missed with device- or self-report aggregate-based measures alone. For example, balance and resistance training are commonly prescribed for people with cancer and survivors; however, these activities are not well captured by device-based measures that may misclassify balance and/or resistance training exercises as light or even sedentary time. Indeed, gym based resistance training exercises were commonly reported using the MARCA at follow up. In this study, MARCA data also enabled a deeper examination and understanding of the feasibility of the text message-enhanced intervention. Mapping of the participants’ strategies to break up sedentary behavior danger zones and tailored physical activity goals with their time use results demonstrated close alignment, with several activities mapping directly onto the most commonly reported sedentary behavior danger zones and MVPA goals. These findings provide further evidence of the success of the enhanced intervention.

The current study has a number of strengths. This is the first study to evaluate a text message-enhanced intervention in people with cancer and survivors that uses a whole-of-day approach, both during and following a supervised clinical exercise rehabilitation program. In combination with self-report methods, it used a gold standard, device-based method for assessing activity; the activPAL monitor. Further, employing a randomized controlled trial design allowed comparison to standard care, and incorporating rigorous assessment of feasibility and acceptability enabled a deeper understanding of the potential scalability of the intervention. Finally, despite the average age of almost 70 years, there were high levels of uptake and engagement in the text message-enhanced intervention, with all eligible participants opting into the intervention. Only one participant was excluded as they did not own a mobile phone, and there was a high reply rate to text messages that invited a response (78%), thus demonstrating that technology-based interventions are feasible and acceptable for this population.

There are several limitations of this study. The relatively small sample size provides only preliminary evidence of the efficacy of the enhanced intervention. A larger study, with statistical power to detect changes in both sitting time and physical activity, is required to confirm the effectiveness of the intervention. While powered for medium effect sizes (considered in terms of d = 0.5), confidence intervals around non-significant effects sometimes failed to rule out potentially large differences between groups of an hour or more. Despite the high levels of feasibility and acceptability, four participants (22%) opted out of receiving text messages at the end of the supervised phase (i.e. 4 weeks), indicating that it did not suit all participants. Reasons provided for opting out included: being sufficiently internally motivated to maintain their goals; not finding the text messages useful; and not liking the directive tone of the text messages. In future iterations, participants should be given the flexibility to opt in and out of extended contact interventions as they develop confidence to self-regulate their behavior. Further, the tone of the text message language should also be tailored based on participant preference, where possible. Patients and survivors of colorectal, breast and prostate cancer volunteered to participate in this study. Whilst these are the three most commonly diagnosed cancers in Australia [47], and thus representative of most Australian exercise oncology interventions, whether the results may have differed in other oncological populations is unknown. There were large changes in computer work, however the MARCA does not collect information pertaining to the purpose of the computer work (e.g. social or for paid or unpaid work). Whether there may have been any unintended consequences on social interaction or work productivity from the study should be explored in future iterations. Finally, at baseline, participants who volunteered for this study had high levels of sitting time (> 8 h per day), but on average were already meeting MVPA guidelines [48] and findings may differ in a less active population.

## Conclusions

In summary, the findings from this study have demonstrated the feasibility, acceptability and efficacy of a text message-enhanced clinical exercise rehabilitation program with a whole-of-day approach to activity behavior change. This novel approach has the potential to extend clinical contact beyond a supervised exercise rehabilitation program, and at scale, while focusing on a broader range of activities and maintaining contact for a longer period of time. These findings may have great impact on future approaches to clinical practice to support people living with and beyond cancer to improve and maintain their health.

## Additional files

Additional file 1: **Table S1.** activPAL protocol. Described activPAL protocol in detail. (DOCX 17 kb)

Additional file 2: **Figure S2.** Heatmap. Example of a heatmap used during coaching sessions derived from activPAL data. (TIFF 1142 kb)

Additional file 3: **Figure S3.** Temporogram used during coaching sessions derived from MARCA data. Example of a temporogram used during coaching sessions derived from MARCA data. (TIFF 1142 kb)

Additional file 4: **Figure S4.** Baseline to 4-week follow-up changes in most frequently reported sedentary behavior and moderate-to-vigorous physical activity activities from the MARCA by duration (min/week) in the text message-enhanced clinic. *Note:* MARCA = Multimedia Activity Recall for Children and Adults. Graph detailing changes in time (min/day) spent in sedentary behaviour and physical activity from baseline to 4-week follow up in the text-message-enhanced clinic. (TIF 1799 kb)

## Abbreviations

AEP: Accredited Exercise Physiologist
CI: Confidence interval
MARCA: Multimedia Activity Recall for Children and Adults
METs: Metabolic equivalents
MVPA: Moderate-to-vigorous physical activity
TV: Television
