# Supplementary material for: Feasibility, acceptability and efficacy of a text message-enhanced clinical exercise rehabilitation intervention for increasing ‘whole-of-day’ activity in people living with and beyond cancer
Source: BMC Public Health. 2019 Jun 3;19(Suppl 2):542. doi: 10.1186/s12889-019-6767-4 (PMC6546618; doi:10.1186/s12889-019-6767-4)
Supplement: Supplementary file 1 — Table S1. activPAL protocol. Described activPAL protocol in detail. (DOCX 17 kb) [file 12889_2019_6767_MOESM1_ESM.docx]

**Table S1.** activPAL protocol

| **Consideration** | **Protocol used** |
| --- | --- |
| Monitor version | activPAL3^TM^ micro |
| Rationale for selecting activPAL monitor | The enhanced clinic targeted whole-of-day behavior changes with an emphasis on reducing sitting. An objective device was required to supplement detailed self-report. |
| Behavioral characteristics of primary interest | The behaviors targeted in the messaging: prolonged sitting time, whole-of-day changes activities across the spectrum from sitting through to moderate-vigorous physical activity. |
| Reliability of the device selected | Inter-device reliability of 0.79 to 0.99 has been reported for the activPAL [33]. |
| Validity information for the activity estimates of interest | The activPAL agrees with direct observation for total time sitting and standing to within a small mean percentage difference of 0.19% (limits of agreement from −0.68% to 1.06%) and 1.4% (limits of agreement from −6.2% to 9.1%), respectively [33]. It measures stepping with <1% error regardless of speed [35] which also equates well with observed walking time −2.0% (limits of agreement −16.1% to 12.1%) [33]. Validation studies support that stepping time can be divided into light and moderate-vigorous with reasonable accuracy. A method using the micro’s summed vector magnitude (SVM) [36] predicts METs with a concordance correlation coefficient of 0.94 relative to portable calorimetry, which is consistent other device-method combinations explored in the same study (ranging from 0.92 for to 0.97) and results in identifying moderate activity with a sensitivity/specificity of 0.955/0.896. Postural transitions are detected accurately relative to direct observations (r=0.97, %bias [95% CI] 0.3(-7.0, +7.7) [34] indicating accumulation measures such as usual bout duration are calculated from valid data. |
| Method and location of monitor attachment | Each device was sealed using a nitrile finger cot and a layer of Opsite then secured to the midline anterior of the participant’s right thigh, atop a small patch of Hypafix which had been applied after alcohol swabbing to minimize any skin irritation. The activPAL was then secured using a transparent film (Tegaderm Roll, 3M). |
| Wear period and number of days | 24 h/d for 7 consecutive days |
| activPAL software version | activPAL Professional Software, v7.2 Research Edition; PAL Technologies Ltd, Glasgow, United Kingdom |
| Settings used: |  |
| • Sampling frequency | 20 Hz (default) |
| • Minimum sitting period | 10 s (default) |
| • Minimum upright period | 10 s (default) |
| Diary data collected and details collected | Wake/sleep, removal times for any removal >30 min |
| Type of file used for data processing | Events files (X, Y, Z version) primarily and 15-s summary files were used to classify stepping intensity |
| Goal for the sampling periods observed | ≥10 h of waking wear time per day *(a priori)* |
| Method(s) for estimating wearing time/removing time in bed/sleep | Events that were ≥50% within self-report waking hours were initially identified as the waking period. The wake time was refined slightly to begin only after the first sitting/lying event of ≥20 min duration at or before the beginning of the initial waking period began. The sleep time was refined to only begins at the last sitting/lying event ≥20 min at or after the end of the initial waking period. Only waking hours were included. Events that were ≥50% within self-reported removal periods were identified as non-wear time and excluded from analysis. |
| Quality control checks implemented | Plots of continuous activity over time were used for checking. Activity during all hours was shown as a line, plotted on top of a blockplot depicting the wear/non-wear classifications, with the diary self-report times annotated alongside the plots. This indicated whether the movement occurring was consistent with having chosen reasonable times (determined subjectively) for transitioning between wake/on and sleep/off, or whether the diary contained errors (e.g. 10 am instead of 10 pm). The method was also used to provide an approximation of the sleep or wake time if it had not been reported (subjectively determined). |
| Action taken for invalid data | Invalid days were excluded. |
| Compliance criteria to define a valid day of observation | Day has ≥10 h of worn waking hours, <95% of time spent in any one behavior (i.e. sitting, standing, or stepping) and ≥500 strides. |
| Number and type of days required for inclusion | ≥1 day of data (any type) to minimize bias from data loss consistent with the CONSORT statement |
| Definition of a day | All time in each event was assigned to the calendar day (midnight-midnight) on which the event began. |
| Data processing package used and methods used to generate key summary variables | Files generated by activPAL software v7.2. SAS version 9.4 was used to perform quality checks and determine valid data.  The MET-level of each 15-second time period was first estimated using the SVM-based method for the activPAL3 micro outlined by Powell et al. [36], then each stepping event was assigned the appropriate MET-level based on the 15-second time period in which it occurred and then classed as light (<3 METs) or moderate-vigorous (≥3 METs) stepping. Time spent in each activity was summed per day, then averaged across valid days. Values were standardized to a 16-hour waking day by multiplying by 16 then dividing by waking wear hours. Usual sitting bout duration was calculated across all sitting/lying bouts occurring during waking wear time on valid days, using the non-linear regression method outlined by Chastin et al. [32]. |
