# Supplementary figures and images for: Feasibility, acceptability and efficacy of a text message-enhanced clinical exercise rehabilitation intervention for increasing ‘whole-of-day’ activity in people living with and beyond cancer
Source: BMC Public Health. 2019 Jun 3;19(Suppl 2):542. doi: 10.1186/s12889-019-6767-4 (PMC6546618; doi:10.1186/s12889-019-6767-4)

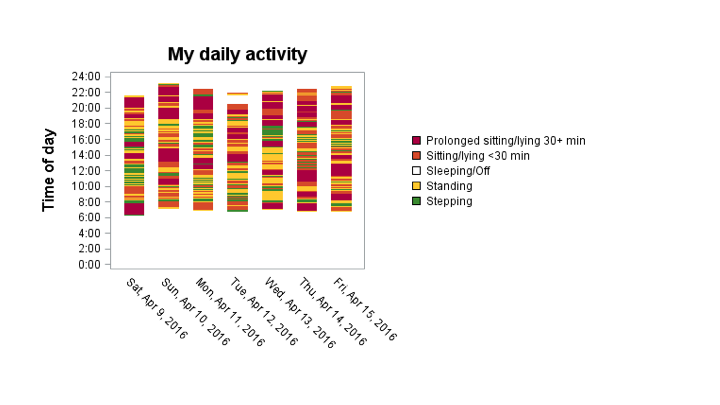

Supplement: Supplementary file 2 — Figure S2. Heatmap. Example of a heatmap used during coaching sessions derived from activPAL data. (TIFF 1142 kb) [file 12889_2019_6767_MOESM2_ESM.tiff]

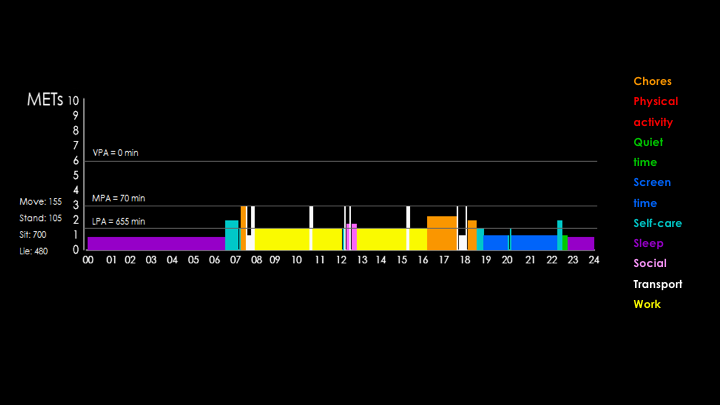

Supplement: Supplementary file 3 — Figure S3. Temporogram used during coaching sessions derived from MARCA data. Example of a temporogram used during coaching sessions derived from MARCA data. (TIFF 1142 kb) [file 12889_2019_6767_MOESM3_ESM.tiff]

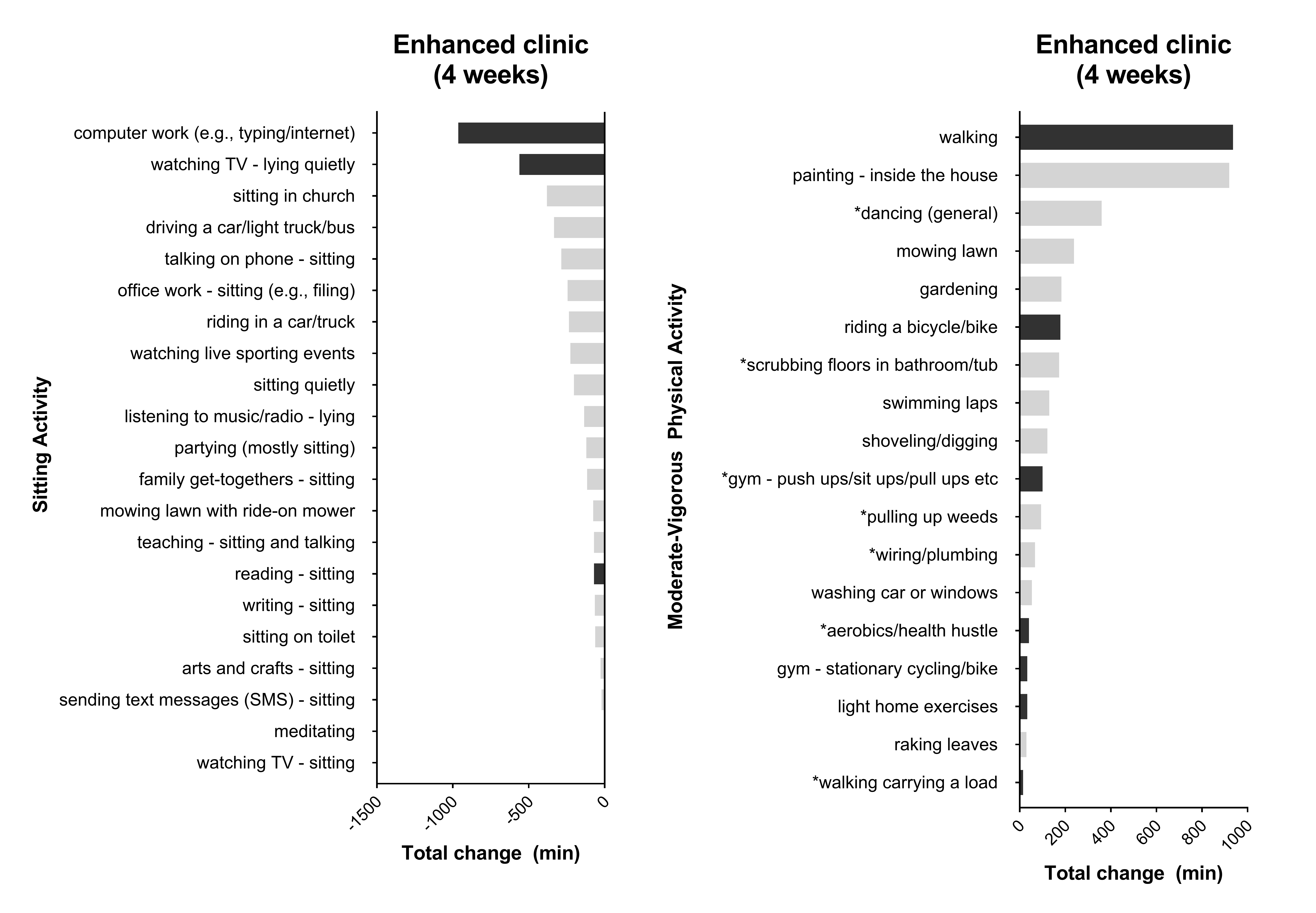

Supplement: Supplementary file 4 — Figure S4. Baseline to 4-week follow-up changes in most frequently reported sedentary behavior and moderate-to-vigorous physical activity activities from the MARCA by duration (min/week) in the text message-enhanced clinic. Note: MARCA = Multimedia Activity Recall for Children and Adults. Graph detailing changes in time (min/day) spent in sedentary behaviour and physical activity from baseline to 4-week follow up in the text-message-enhanced clinic. (TIF 1799 kb) [file 12889_2019_6767_MOESM4_ESM.tif]
